# Increased Adenine Nucleotide Degradation in Skeletal Muscle Atrophy

**DOI:** 10.3390/ijms21010088

**Published:** 2019-12-21

**Authors:** Spencer G. Miller, Paul S. Hafen, Jeffrey J. Brault

**Affiliations:** Indiana Center for Musculoskeletal Health, Indiana University School of Medicine, Department of Anatomy, Cell Biology & Physiology, 635 Barnhill Dr., Van Nuys Medical Science Bldg. 5035, Indianapolis, IN 46202, USA; millespe@iu.edu (S.G.M.); pshafen@iu.edu (P.S.H.)

**Keywords:** atrophy, muscle, adenine nucleotide, ATP, AMP, uric acid, cachexia, sarcopenia, energetics

## Abstract

Adenine nucleotides (AdNs: ATP, ADP, AMP) are essential biological compounds that facilitate many necessary cellular processes by providing chemical energy, mediating intracellular signaling, and regulating protein metabolism and solubilization. A dramatic reduction in total AdNs is observed in atrophic skeletal muscle across numerous disease states and conditions, such as cancer, diabetes, chronic kidney disease, heart failure, COPD, sepsis, muscular dystrophy, denervation, disuse, and sarcopenia. The reduced AdNs in atrophic skeletal muscle are accompanied by increased expression/activities of AdN degrading enzymes and the accumulation of degradation products (IMP, hypoxanthine, xanthine, uric acid), suggesting that the lower AdN content is largely the result of increased nucleotide degradation. Furthermore, this characteristic decrease of AdNs suggests that increased nucleotide degradation contributes to the general pathophysiology of skeletal muscle atrophy. In view of the numerous energetic, and non-energetic, roles of AdNs in skeletal muscle, investigations into the physiological consequences of AdN degradation may provide valuable insight into the mechanisms of muscle atrophy.

## 1. Introduction

Skeletal muscle wasting (atrophy), which occurs with chronic disease, inactivity, or aging, is a devastating condition that can lead to reduced muscle function and quality of life. Muscle atrophy is defined as the loss of skeletal muscle mass and is associated with a loss of muscle fiber cross sectional area and muscle protein content. The pathogenesis and signaling pathways controlling muscle atrophy have been reviewed extensively elsewhere [1,2,3]. In this review, we will focus on a topic that has been largely overlooked in muscle atrophy, adenine nucleotide content. We will present evidence that a decrease in concentration of adenine nucleotides (AdNs: ATP, ADP, AMP), especially ATP, is a common feature of skeletal muscle atrophy. Furthermore, this drop in the AdNs appears to reflect the induction of enzymes that actively degrade AdNs, rather than the loss of ATP regenerative capacity.

## 2. AdNs in Skeletal Muscle

The AdNs are organic compounds composed of adenine (purine base), ribose (5-carbon sugar), and 1-3 phosphate groups. In humans, skeletal muscle AdN content is often reported after adjusting for muscle dry weight, with normal resting concentrations ([ATP], [ADP], [AMP]) near 21–24 mmol·kg^−1^ for [ATP], 3–4 mmol·kg^−1^ for [ADP], and 0.07–0.10 mmol·kg^−1^ for [AMP] [4,5,6]. Importantly, ATP content differs by fiber type, with human fast-twitch fibers (~23 mmol·kg^−1^) having greater [ATP] compared to slow-twitch fibers (~21 mmol·kg^−1^) [4]. The fiber-type differences in [ATP] are even more pronounced in rodent skeletal muscle. For example, [ATP] is 7–8 µmol·g^−1^ in rodent fast-twitch fibers and is 4–5 µmol·g^−1^ in slow-twitch fibers. Resting [ADP] and [AMP] are comparable between fiber types, ranging between 0.8 to 1.1 µmol·g^−1^, and 0.028 to 0.032 µmol·g^−1^, respectively [7,8,9].

ATP diffuses freely throughout the cytosol (i.e., it is unbound), while the vast majority of ADP and AMP are bound to intracellular proteins (such as actin) and are therefore not free to diffuse or react with other proteins [8]. While the large disparity between free concentrations of ATP and ADP gives rise to the enormous energy potential of ATP hydrolysis (ΔG_ATP_) [10], it is also important to note that the AdNs are involved in many other essential physiological processes.

### 2.1. Immediate Energy Source for Contraction

The most well-appreciated role of ATP in skeletal muscle is that it provides the immediate energy necessary for muscle contraction. The major ATPases consuming energy during muscle contraction are: sarco/endoplasmic reticulum Ca^2+^-ATPase (SERCA) [11], myosin ATPase [12], and Na^+^/K^+^-ATPase [13]. Together, these enzymes are responsible for nearly all of the increased ATP turnover observed during muscle contractions [14]. Intracellular [ATP] is buffered during acute periods of increased energetic demands through the creatine kinase reaction (PCr + ADP ↔ Cr + ATP). Thus, conditions affecting total AdN content and/or basal [ATP] may also impact these energetic buffering processes [15].

### 2.2. Signaling Molecules

ATP can function as a direct cellular signal, or a structural base for secondary messengers. A well-established example of AdNs in cellular signaling is the direct regulation of the 5′ AMP-activated protein kinase (AMPK). Specifically, competitive binding between ATP and AMP allows AMPK to respond to fluctuations in energy homeostasis and impacts its interaction with other proteins [16,17]. When the ATP:AMP ratio increases, and more ATP is inexorably bound to AMPK, the kinase becomes more susceptible to dephosphorylation and deactivation [18]. As AMPK is capable of directly affecting signaling processes related to protein synthesis [19], mitochondrial biogenesis [18,20], and substrate uptake and oxidation [21], conditions leading to alterations in AdN content could have broad structural and metabolic implications in skeletal muscle.

ATP also provides the structural base for the formation of the secondary messenger cyclic AMP (cAMP). For the formation of cAMP, the enzyme adenylate cyclase removes two of the phosphate groups from ATP and creates a cyclic bond arrangement between the existing sugar and phosphate group [22]. cAMP is then involved in many processes related to gene transcription [23], ion channel regulation [24], and metabolism [25].

### 2.3. RNA/DNA/Protein Metabolism

While the ATP energy requirements for protein metabolism are generally considered quite high [26], ATP hydrolysis, per se, is not required for all aspects of protein synthesis and/or degradation. For example, non-hydrolyzing ATP analogs lead to the elongation of short gene transcripts [27] and are sufficient to maintain maximal transcription rates via RNA polymerase II [28]. Furthermore, while the majority of protein degradation is carried out by the 26S proteasome, which regulates proteolysis largely via ATP-hydrolyzing pathways [29], ATP binding, but not hydrolysis, is essential for proper structural alignment between the proteasomal ATPases and the core particle [30].

### 2.4. Protein Solubility

Hydrotropes are molecular compounds that solubilize hydrophobic substances, such as proteins, in aqueous solutions. This may be especially important in muscle cells to prevent aggregation of the massive quantities of myofibrillar proteins in the cytoplasm [31]. ATP within normal physiological ranges (5–10 mM) displays hydrotropic characteristics, preventing protein aggregation and liquid-liquid phase separation. Importantly, these effects do not appear to be dependent on ATP-hydrolysis as they persist even when using a non-hydrolyzing ATP analog (APPNP) [32].

### 2.5. Co-Factor Synthesis

AdNs also influence cell metabolism through both direct and indirect control of major metabolic pathways. For example, AdNs can directly regulate glucose and glycogen metabolism by allosteric regulation of enzymes such as phosphofructokinase-1, and glycogen phosphorylase [33,34]. Moreover, AdNs indirectly regulate metabolism by controlling the synthesis of necessary co-factors such as coenzyme-A [35], flavin adenine dinucleotide [36], and nicotinamide adenine dinucleotide (NAD+) [37].

Reductions in ATP and NAD+ levels have been found in muscles during aging [38], and reducing ATP levels in vitro leads to linear decreases in NAD+ [39]. NAD+ synthesis requires ATP and nicotinamide mononucleotide (NMN), and NMN synthesis requires phosphoribosyl pyrophosphate (PRPP), which is also used for de novo purine synthesis and purine salvage [40]. Therefore, lower ATP levels may also reduce NAD+ synthesis rates due to limited ATP availability and/or increased competition for PRPP between pathways for adenine nucleotide and NAD+ synthesis.

## 3. AdN Metabolism

Adult skeletal muscle can synthesize nucleotides through both de novo and salvage pathways [40,41]. The de novo pathway involves assembling a purine base onto a ribose-5-phosphate molecule, while salvage pathways attach a ribose-5-phosphate molecule to an existing purine ring. Purine synthesis rates through both pathways are limited by the availability of PRPP, which supplies the ribose-5-phosphate molecule, and feedback inhibition by their purine monophosphate end products [40]. Both pathways result in the formation of inosine 5’monophosphate (IMP).

**Figure 1:**
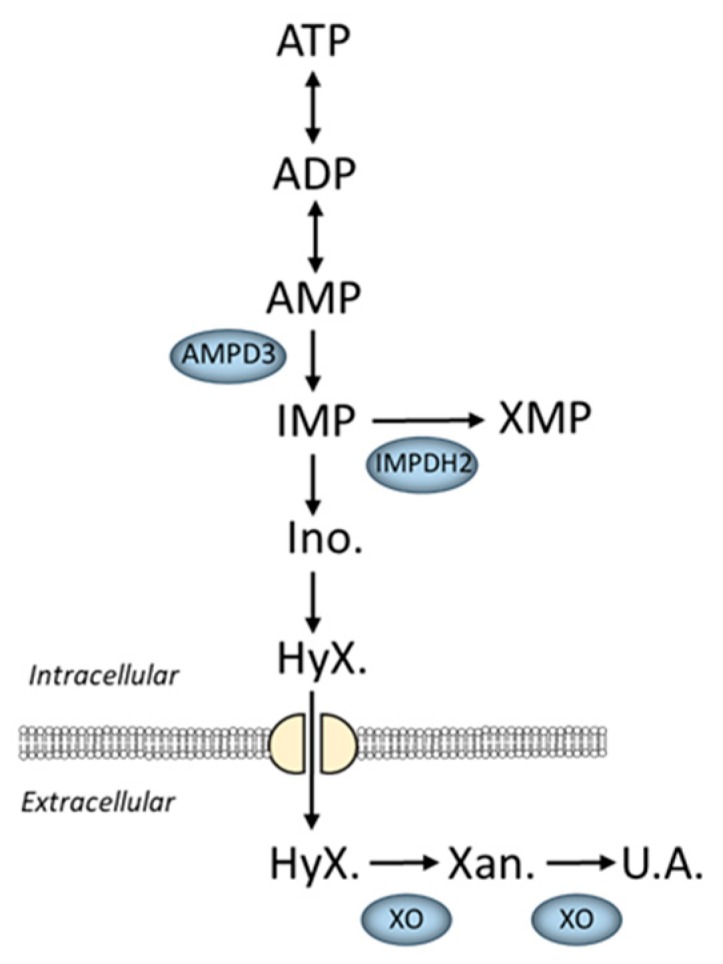
Schematic of adenine nucleotide degradation in skeletal muscle. Enzymes in blue have increased expression and/or activity in various models of skeletal muscle atrophy. Abbreviations: AMPD3, AMP deaminase 3; IMPDH2, IMP dehydrogenase 2; XO, xanthine oxidase; Ino., inosine; HyX., hypoxanthine; Xan., xanthine; U.A., uric acid.

Skeletal muscle also has pathways that regulate AdN degradation (Figure 1). The degradation of AdNs is evident in conditions where ATP hydrolysis outpaces the rate of ADP re-phosphorylation, such as during intense contractions [42] or hypoxia [43,44]. Initially, adenylate kinase catalyzes the transfer of a terminal phosphate from one ADP molecule to another, producing ATP and AMP (ADP + ADP ↔ ATP + AMP). Next, AMP can either be dephosphorylated by 5’nucleotidase (5’NT: AMP → adenosine + P_i_) or deaminated by AMP deaminase (AMPD: AMP → IMP + NH_3_), with AMPD being the predominant route in skeletal muscle [45]. The AMPD reaction in muscle is thermodynamically irreversible, which, in combination with the near equilibrium of adenylate kinase, results in a preservation of ΔG_ATP_ at the expense of degrading the total nucleotide pool (ATP+ADP+AMP) [10].

It is important to note that the AdN degradation products—purine nucleosides (i.e., inosine, adenosine) and bases (i.e., hypoxanthine, adenine)—can be lost from muscle due to transport and/or diffusion across cell membranes [46,47,48,49]. When these purine catabolites are released from muscle, they can be taken up by various other cell types, such as liver and endothelial cells [49,50,51], and further degraded by xanthine oxidase, which converts hypoxanthine to xanthine to uric acid [50]. Importantly, xanthine oxidase is not found in skeletal muscle [51,52]. In most mammals, aside from humans and some primates, uric acid can be further degraded to allantoin by the enzyme urate oxidase [53]. Thus, uric acid is the terminal end product of nucleotide catabolism in humans, while allantoin is the end product in rodents.

## 4. AdNs in Disease-Associated Muscle Atrophy

Muscle atrophy and loss of muscle protein during chronic diseases are mediated by a host of factors including: elevated pro-inflammatory cytokines, tumor derived factors, cell derived cytokines, glucocorticoids, endotoxins, and/or, decreased anabolic hormones, resistance to anabolic stimuli, anorexia, and reduced physical activity [54,55,56,57]. As outlined in this section, AdNs are decreased in atrophied skeletal muscle, regardless of the disease or precise factor(s) that induce muscle loss.

### 4.1. Cancer

The weakness and wasting (cachexia) of skeletal muscle in cancer is well-established. Cachexia is estimated to effect approximately half of all cancer patients and accounts for 20% of all cancer related deaths [58]. The severity of muscle atrophy during cancer varies with tumor type, size, and location [59], but maintaining muscle mass and function during cancer can significantly improve chemotherapy tolerance, quality of life, and prolong lifespan [57,60].

In gastric carcinoma patients, biopsies from the quadricep femoris showed significantly less ATP, ADP, AMP, and total AdN contents compared to controls [61]. While interpretations in such human studies may be complicated by decreased nutrient intake, decreased AdNs in cancer cachexia have been validated in rat cancer models using pair-feeding to demonstrate that reductions in [ATP] in cancer cachexia appear to be independent of food intake [62]. In another rodent model of cancer cachexia, Hochwald et al. [63] measured 20% lower [ATP] and 30% lower [ADP] in gastrocnemius muscles collected 19 days after soft-tissue sarcoma cell injection, relative to pair fed controls. Moreover, 10 days after tumor resection, muscle [ATP] returned to that of non-tumor bearing controls.

The oxidation of hypoxanthine to uric acid may play a role in the development of cancer cachexia. This has become evident through the observations that xanthine oxidase inhibitors, such as allopurinol or febuxostat, can attenuate muscle wasting and increase survival in rodent models of cancer cachexia [64,65]. Treating mice with these inhibitors lowers circulating uric acid and associated markers of oxidative stress, while reducing markers of skeletal muscle protein degradation [64].

### 4.2. Diabetes

Muscle atrophy and decreased muscle function is common among patients with type II diabetes, especially in older populations. Moreover, muscle atrophy is thought to cause a vicious cycle in which the loss of strength and endurance leads to heightened incidence of falls, hospitalization, and sedentary behavior, which subsequently leads to more muscle atrophy and so on [66].

In human patients with type II diabetes, ATP and ADP were measured in vivo in vastus lateralis [67]. Compared to age, sex, and BMI matched non-diabetic controls, diabetic patients display significantly less ATP, ADP, and PCr (phosphocreatine). Importantly, these authors used a combination of muscle biopsy (for ATP) and in vivo ^31^P-magnetic resonance spectroscopy (MRS). This study is unique, as the absolute concentrations of ATP from muscle biopsies were used to calculate the ^31^P-MRS values. Traditionally, ATP is assumed to be constant among subjects [68], and thus, MRS studies would not be able to reveal true differences in ATP. Ultimately, Ripley et al. [67] found that diabetic patients do indeed display significant reductions of ATP and ADP content compared to healthy controls.

In db/db mice, a mouse model of type II diabetes mellitus, metabolomics analysis of atrophied quadricep femoris muscles revealed significantly greater amounts of AdN degradation products, such as IMP, hypoxanthine, adenosine, and uric acid than muscles of non-diabetic controls [69]. After 5 weeks of moderate intensity treadmill running in db/db mice, the concentrations of these degradation products returned to lower levels and quadricep muscle mass was recovered.

### 4.3. Chronic Kidney Disease

Muscle atrophy and weakness is highly prevalent in patients with chronic kidney disease, which is defined as any general condition that decreases kidney function [70]. The reduced skeletal muscle mass and functional capacity that accompany chronic kidney disease lead to increased morbidity and mortality in affected adults [71].

Reductions in kidney function result in an increase in circulating uremic toxins, which are normally eliminated by properly functioning kidneys. One of the first studies to examine the effects of uremia on skeletal muscle AdNs and phosphocreatine was conducted by Del Canale et al. [72]. In their study, muscle biopsies were taken from the lateral quadriceps femoris muscle of non-dialyzed patients suffering from end-stage renal failure. Compared to age matched healthy controls, uremic patients had approximately 15% less ATP, PCr, and total AdNs than controls, without any observable changes in ADP, AMP, or total creatine. The reduction in ATP resulted in a decrease of the ATP-associated energetic ratios (ATP:ADP, ATP:AMP) and a drop in apparent cellular energy charge [72].

Well-controlled in vitro studies have begun to elucidate the impact of uremic toxins on skeletal muscle energetics. Sato et al. [73] performed metabolomic analysis of C2C12 myotubes treated with indoxyl sulfate, a uremic toxin. Treated myotubes displayed significantly lower [ATP] in conjunction with the accumulation of IMP, hypoxanthine, and xanthine [73].

### 4.4. Heart Failure

Reduced work capacity in patients with chronic heart failure is the result of reduced circulatory capacity and increased skeletal muscle atrophy [74,75]. Muscle biopsies from the quadricep femoris of elderly patients with chronic congestive heart failure were compared to that of healthy, age-matched controls. The skeletal muscle from heart failure patients had 17% lower [ATP] [76]. These studies also found that 8 weeks of either dietary supplementation or treatment with the ACE inhibitor enalapril were unable to restore [ATP] in heart failure patients [77].

Though the regulation of skeletal muscle AdNs in heart failure has not been extensively studied, there is some evidence in healthy populations that serum uric acid (an end-product of nucleotide degradation) is partly controlled by its production in skeletal muscle [78]. Thus, it is possible that elevations of circulating uric acid, or hyperuricemia in heart failure [79], may reflect increases in AdN degradation in skeletal muscle. Interestingly, the positive outcomes associated with lower uric acid levels appear to be more dependent on the actual inhibition of xanthine oxidase, as opposed to absolute changes in serum uric acid levels [80].

### 4.5. Chronic Obstructive Pulmonary Disease (COPD)

The pathology of COPD is not limited to decreased respiratory function in the lungs; systemic effects such as altered hormonal status and increased skeletal muscle atrophy are characteristic of COPD [81,82]. Steiner et al. [83] observed that skeletal muscle of COPD patients had 20% less ATP and a lower ATP/AMP ratio. In a study of COPD patients experiencing acute respiratory failure, patients had 30% less ATP and reduced total AdNs in skeletal muscle [84].

In contrast, tibialis anterior muscle biopsies of COPD patients revealed no statistically significant differences in the concentrations of ATP, ADP, AMP, or total AdNs between patients and healthy, age-matched controls. However, they did observe a significantly lower ATP/ADP ratio and significantly greater [IMP] in the COPD patients, suggesting increased flux through AdN degradation pathways during COPD [85].

### 4.6. Intensive Care/Sepsis

Severe systemic inflammation and multi-organ failure are pathological features of acute critical illnesses and require intensive care unit treatment. Rapid muscle atrophy and muscle weakness during intensive care contribute significantly to patient morbidity and mortality as well as long term recovery from critical illness [86].

In an early study of the critically ill, patients were separated into two groups: (1) those admitted due to an acute critical illness caused by circulatory or respiratory insufficiency or, (2) those admitted due to a prolonged chronic disease. Both groups had significantly less [ATP] and total AdNs relative to control values [87]. In agreement, Puthucheary et al. [88] observed that critically ill patients had significantly lower [ATP] and [PCr] than healthy subjects. Additionally, the timing of sampling was important. Within a day of admittance to intensive care, critically patients with a chronic disease (COPD, ischemic heart disease, hypertension, and liver cirrhosis being the most common) had significantly less ATP than those without a chronic disease. After 7 days, reductions in [ATP] were similar between critically ill patients with, and without, pre-existing chronic disease. Unfortunately, [ADP], [AMP], and [IMP] were not reported [88].

Experimental rodent models used to study the effects of sepsis have also shown decreased skeletal muscle [ATP] and total AdNs following cecal ligation and puncture [89,90], intraperitoneal fecal-agar pellet [91], and lipopolysaccharide injections [92]. Together, these studies in both humans and rodents indicate that chronic diseases/infections can result in a significant decrease in resting [ATP] and the total AdN pool in skeletal muscle.

### 4.7. Muscular Dystrophy

The muscular dystrophies are a group of genetic diseases characterized by progressive muscle wasting and weakness. These diseases are caused by a variety of genetic defects affecting proteins that are essential for the proper stabilization and function of the muscle cell membrane, or sarcolemma [93]. The compromised sarcolemma in muscular dystrophy results in a state of chronic muscle damage clinically manifested through elevations in serum creatine kinase and pyruvate kinase [94]. In addition, histological analysis of dystrophic muscle shows increased muscle necrosis [95], macrophage infiltration [96], and lipid/connective tissue deposits [97].

Duchenne muscular dystrophy (DMD), the most common type of dystrophy in humans, presents with the most severe symptoms and is the most extensively studied type of muscular dystrophy [93]. In DMD patients, skeletal muscle [ATP] and total AdNs are severely reduced (by up to ~50%) [98,99,100,101,102,103]. Interestingly, investigations into the reduced [ATP] have uncovered extreme mitochondrial myopathies in DMD, thus expanding our recognition of DMD as a metabolic myopathy [104,105]. While mitochondrial dysfunction undoubtedly contributes to the reductions in [ATP], the concomitant reductions in total AdNs, in excess of what is seen in other atrophies or even extreme exercise, suggest that additional mechanisms might contribute to the energetic deficits. For example, increases in purine excretion in the urine of DMD patients suggests increased AdN turnover (degradation) [106]. Also, treatments aimed at increasing intracellular [ATP] in DMD muscle, either by stimulating purine salvage (adenylosuccinic acid; ASA) or by inhibiting purine breakdown (allopurinol), lead to positive outcomes such as improved muscular strength [100,104] and reduced lipid deposits [107]. However, it is not clear to what extent the nucleotide pool is enzymatically degraded (Figure 1) or merely lost by diffusion out of the cell due to chronic damage/disruption of the sarcolemma.

## 5. AdNs in Denervation Atrophy

Loss of innervation to skeletal muscle can occur as the result of spinal cord injury or damage of isolated nerves affecting specific muscle groups. Spinal cord injury can reduce skeletal muscle mass by 30–45% in as little as 6 weeks, [108,109]. While the regulation of AdNs in human skeletal muscle following spinal cord injury have not been characterized, the surgical protocols employed in animal research have provided an excellent opportunity to better understand the regulation of AdNs. In rats, for example, surgical denervation (sectioning of the sciatic nerve) of the gastrocnemius muscle reduces [ATP] as early as 5 days post-surgery [110] with further drops of 25% to 45% by 4 weeks post-surgery [111,112].

## 6. AdNs in Disuse Atrophy

Skeletal muscle disuse is a broadly used term that encompasses reductions in mechanical loading and muscle activity such as spaceflight, bed rest, limb immobilization, and limb suspension [113]. The negative effects traditionally associated with disuse are decreased myofiber cross sectional area, reduced force producing capacity, and increased fatigability. 

In humans, several early studies investigating the effect of limb immobilization on skeletal muscle [ATP] reported no changes [114,115]. In these studies, muscle samples were frozen in liquid nitrogen, as opposed to being rapidly freeze-clamped. Since skeletal muscle collection/freezing techniques differentially induce ATP hydrolysis, the methods used to ‘quick-freeze’ the tissue may add to the variability in AdN measures [8,116]. Nonetheless, recent investigations suggest that inhibition of AdN degrading enzymes can attenuate skeletal muscle atrophy in humans. Specifically, the administration of allopurinol to inhibit xanthine oxidase—which regulates purine degradation through the hypoxanthine-xanthine-uric acid axis—attenuates human skeletal muscle atrophy following two weeks of immobilization [117]. 

Evidence of increased AdN degradation is supported by well-controlled animal disuse studies. For example, hindlimb unloading via tail suspension, which unloads the hindlimbs while allowing movement of the limb, leads to progressive declines in muscle [ATP] after 7 days (−9%) and 14 days (−26%) of disuse [112]. Similarly, muscle disuse brought on by the combination of tail suspension (mechanical unloading) and immobilization via foot casting (fixed muscle length) leads to decreases in muscle [ATP] [118] and total AdNs [119]. In addition, products of purine degradation, such as hypoxanthine, xanthine, and urate—all products of purine degradation—are increased 2- to 3-fold after 12 days of cast immobilization [120]. Furthermore, the use of allopurinol, similar to what was observed with oral administration in human disuse models, maintains skeletal muscle contractile function [121] and mass [122] in unloaded rodent skeletal muscle.

## 7. AdNs in Aging-Associated Atrophy

The loss of skeletal muscle mass and function with advancing age—termed sarcopenia—is a major contributor to physical impairment and disability [123,124]. Muscle mass begins to decline after age 30, with substantial reductions (−10%) by age 50. After age 50, the rate of reduction further increases as total muscle mass loss approaches 35–40% by 80+ years of age [125,126]. These decreases are mainly attributed to reductions in the average size of type II (fast) fibers, while type I (slow) fibers change to a lesser extent [127,128].

There is strong evidence that AdN degradation increases with age, potentially contributing to the pathology, albeit with some conflicting reports. For example, clear age-related reductions in the total AdN pool and [ATP] [129,130,131,132] have been reported in human skeletal muscle. On the contrary, others show no statistical differences in total AdNs or [ATP] when comparing young versus aged muscle [133,134]. One possible explanation for this discrepancy may be due to the grouping of continuous variables (age) common in clinical based research. Such dichotomization severely compromises a study’s statistical power [135,136] and decreases the likelihood of detecting subtle, yet potentially meaningful, differences. Case in point, though Conley et al. [129] found no group differences in [ATP] between groups, when the data were linearized according to age, there was a statistically significant age-related decline in skeletal muscle [ATP] at a rate of nearly 1% per year (−0.048 mM ATP/year). In support of age-induced reductions of the AdN pool and [ATP], significant increases in circulating AdN degradation products are characteristic of human sarcopenia [137,138,139].

Carefully designed and controlled animal aging studies have been instrumental for further identification of the age-related changes in the regulation of ATP and AdNs. Increased AdN degradation in aging skeletal muscle is supported by complementary reports of decreased [ATP] [140,141,142], reductions in the total AdN pool [143,144], and increased formation of purine degradation products such as hypoxanthine [145], and ammonia [146].

## 8. Potential Mechanisms

The data reviewed in the previous sections demonstrate that a measurable reduction in ATP with small reductions, or even no changes, in ADP and AMP levels occur in atrophic skeletal muscle. This is in stark contrast to the drop in ATP that occurs during a mismatch in energy production versus energy demand (e.g., intense contractions, hypoxia, or mitochondrial defects), where a drop in ATP is accompanied by an increase in ADP and/or AMP [6,147,148]. Therefore, these data suggest that the fall in ATP during atrophy is not strictly due to a mismatch in energy supply/demand. We propose that this reduction in ATP is the result of increased rates of AdN degradation. This is supported by studies demonstrating the induction of three major nucleotide degrading enzymes in atrophying muscle: AMP deaminase 3 (AMPD3), IMP dehydrogenase 2 (IMPDH2), and xanthine oxidase (Figure 1).

First, AMPD3 is a cytosolic enzyme that mediates the thermodynamically irreversible deamination of AMP to IMP. AMPD3 mRNA is reported to be one of the most upregulated genes in atrophying muscles from cancer [149], diabetes [149], renal failure [150], aging [151], Huntington’s disease [152], fasting [149,153], unloading [154], and denervation [153,155,156]. However, AMPD3 expression has not been reported to increase in muscular dystrophy. The increase in AMPD3, when present, occurs early in the progression of atrophy at times when protein degradation rates are highest [149,153]. There is also evidence of increased AMPD3 substrate affinity and maximal activity in denervation atrophy [155,156], further supporting the importance of this enzyme in atrophy.

Second, expression of IMPDH2, which degrades the product of the AMPD reaction (IMP), is also highly induced under numerous atrophic conditions [149,157]. By removing the product of the AMPD reaction, IMPDH2 may favor more rapid degradation of AMP during atrophy.

Third, inhibition of xanthine oxidase, which degrades hypoxanthine to xanthine to uric acid, protects against muscle mass loss [117]. The protective effects of xanthine oxidase inhibition, such as with allopurinol, have been attributed to reductions in ROS concentrations and activation of oxidative stress pathways that suppress protein synthesis and/or increase protein degradation [64,122,158,159]. However, while inhibition of xanthine oxidase decreases ROS production by preventing hypoxanthine oxidation, it is still unknown whether the protective effects may be partly due to concomitant slowing of adenine nucleotide degradation.

## 9. Conclusions

A reduction in the concentration of ATP and the total AdN pool is a common feature of atrophic muscles in a large variety of human diseases and clinical conditions. This reduction is seemingly caused by increased nucleotide degradation, based on observations of increased activities and products of AdN degrading enzymes. The inhibition of degrading enzymes, such as xanthine oxidase, appears capable of partially preserving muscle mass, enhancing muscle mass recovery, and prolonging lifespan under atrophic conditions. The use of such inhibitors would be expected to prevent both (1) the generation of reactive oxygen species and, (2) nucleotide degradation. The extent to which each of these inhibitory effects, either independently or in conjunction, impact skeletal muscle atrophy has not been established. Furthermore, given the numerous energetic, and non-energetic, roles of AdNs in skeletal muscle, investigations into the physiological consequences of AdN degradation on these processes may provide valuable insight into the mechanisms of muscle atrophy.

## Abbreviations

ATP	adenosine triphosphate
ADP	adenosine diphosphate
AMP	adenosine monophosphate
IMP	inosine monophosphate
AdN	adenine nucleotide
PCr	Phosphocreatine
cAMP	cyclic AMP
AMPD3	AMP deaminase 3
IMPDH2	inosine monophosphate dehydrogenase 2
COPD	chronic obstructive pulmonary disease
